# Etiological, clinical, and laboratory evaluation of congenital hypothyroidism and determination of levothyroxine (LT4) dose at treatment interruption in differentiating permanent vs. transient patients

**DOI:** 10.55730/1300-0144.5533

**Published:** 2022-07-24

**Authors:** İsmail DÜNDAR, Mehmet Akif BÜYÜKAVCI, Nurdan ÇİFTÇİ

**Affiliations:** 1 Department of Pediatric Endocrinology, Faculty of Medicine, İnönü University, Malatya, Turkey; 2 Department of Developmental Pediatrics, Faculty of Medicine, İnönü University, Malatya, Turkey; 3 Department of Pediatric Endocrinology, Malatya Training and Research Hospital, Malatya, Turkey

**Keywords:** Congenital hypothyroidism, transient hypothyroidism, permanent hypothyroidism, etiology

## Abstract

**Background/aim:**

Congenital hypothyroidism (CH) is the most common cause of preventable but irreversible mental retardation in children, although the risk has been widely abolished by national neonatal screening programs. The aim of this study was to determine, (a) the cause of CH, (b) the etiological cause of persistent CH and (c) to investigate the role of laboratory and clinical data in predicting persistent and transient CH.

**Materials and methods:**

Patients diagnosed with CH, who started L-thyroxine treatment and were followed up for at least three years were included. Patient data were reviewed retrospectively. Serum thyroid hormones were measured four weeks after discontinuation of therapy at age three or earlier. Cases with a thyroid-stimulating hormone (TSH) value of >10 mIU/mL were accepted as permanent hypothyroidism, while cases with normal TSH values for six months after cessation were accepted as transient hypothyroidism.

**Results:**

There were 232 treated cases, of whom 108 (46.6%) were female, and 169 (72.8%) were eventually diagnosed with transient CH. The best cut-off point for predicting permanent status was determined as LT4 cut-off dose ≥1.45 mcg/kg/day. The median (range) duration of L-thyroxine treatment in transient hypothyroid cases was 24 (range: 6–36) months, and treatment was discontinued before the age of three years in 64%.

**Conclusion:**

It has been shown that the most common etiologic cause of CH is transient hypothyroidism. The thyroxine dose at the time of discontinuation of treatment and free thyroxine and TSH levels at the time of diagnosis are essential determinants in distinguishing permanent/transient CH.

## 1. Introduction

Congenital hypothyroidism (CH) is defined as insufficient thyroid hormone production as a result of dysfunction of the hypothalamic-pituitary-thyroid (HPT) axis present at birth [1]. Thyroid hormone affects almost every organ system, playing a crucial role in normal growth and neurological development. CH affects approximately 1:2000 newborns, and, if not detected and treated early and effectively, can have devastating effects on neurocognitive development [2]. There has been a widespread introduction of national neonatal screening programs for the early detection and rapid diagnosis of CH, as appropriate replacement treatment with sodium levothyroxine (LT4) is effective in abolishing most of the deleterious effects of lack of endogenous thyroid hormone [3].

With screening programs in developed countries, babies with CH are diagnosed and treated without delay. However, CH continues to be a significant cause of mental retardation in countries that do not have a screening program [4]. Depending on the underlying cause, patient suspected of having CH may be transient or permanent. While permanent cases due to thyroid gland dysgenesis or defects in thyroidal enzyme systems that cause dyshormonogenesis, constituted more than 75%–80% of all patients before the introduction of screening programs or in the earliest periods of screening, the frequency of cases with transient CH has gradually increased. The widespread use of screening programs and a tendency to reduce cut-off levels of capillary thyroid-stimulating hormone (TSH) that will trigger referral for formal thyroid assessment, has meant that neonates with lower blood-spot TSH levels are increasingly being referred [5]. While the frequency of CH in regions with screening was one in 2000–3000 live births, it was reported that this rate was one in 6700 live births before screening [6].

Determining the etiology of raised neonatal TSH is important in terms of the duration of the treatment [7]. Permanent CH is often caused by thyroid dysgenesis or dyshormonogenesis, and in these patients, lifelong thyroid hormone therapy should be continued [1,5]. The etiology of transient elevated TSH is multifactorial. Although there are situations in which treatment can be terminated earlier in transient CH, it is recommended that these patients be treated up to three years of age to protect the majority of neurodevelopment which occurs in this period and is thyroid-hormone dependent and then assessed subsequently [1,5,8,9].

While the number of referrals from screening programs increased markedly as TSH threshold values decreased, the incidence of permanent CH did not change much [8,10]. For this reason, developing criteria for the distinction between transient elevated neonatal TSH and permanent CH is essential. The aims of this study were: (1) to determine the frequency of transient and permanent hypothyroidism in cases diagnosed with CH in our clinic; (2) to determine the etiology in cases with permanent and transient CH; (3) and to reduce unnecessary treatment in transient CH by determining possible parameters that will help to distinguish between permanent and transient CH.

## 2. Materials and methods

Medical records of patients diagnosed sequentially with CH in the pediatric endocrinology clinic between January 2011 and July 2020 were retrospectively reviewed. Cases diagnosed with CH in the newborn and infancy periods, started on LT4 treatment, and reached at least three years old were included. At the same time, patients who started treatment in other centers, whose thyroid function tests were known at the time of diagnosis and during follow-up, and who were followed up regularly in our clinic were also included in the study. Exclusion criteria were: (1) patients diagnosed at another center and undergoing LT4 replacement but lacking data on initial LT4 dose and/or pretreatment thyroid function tests; (2) patients not followed up regularly; and (3) follow-up of patients for less than 3 years.

The age in days, sex, age at diagnosis, age at start of treatment, consanguinity between parents, gestational week at delivery, birth weight, family history of hypothyroidism, iodine exposure, maternal history of antithyroid drug use, free thyroxine (fT4) at diagnosis, free triiodothyronine (fT3), serum TSH levels (at diagnosis), and diagnostic thyroid ultrasonography (USG) results of the cases were recorded retrospectively from their files. Additionally, height SDS (standard deviation score), weight SDS, body mass index (BMI), BMI SDS, and head circumference SDS data were recorded during diagnosis and follow-up. The data of the initial and follow-up LT4 doses of the cases were recorded. The diagnosis of CH was made following the Consensus of the European Society of Pediatric Endocrinology, and LT4 treatment was started for the diagnosed patients [11]. In patients with low-normal serum fT4 and high serum TSH at the time of diagnosis, treatment was started at 2.5–15 mcg/kg/day, and the LT4 dose was adjusted according to serum fT4 and TSH levels.

Patients with TSH level <5.6 mIU/mL in the follow-up and thyroid gland in USG were discontinued at age three years or earlier. Cases with agenesis or ectopia detected on USG were accepted as permanent CH, and LT4 treatments were not discontinued. Serum thyroid hormones were measured 4–6 weeks after LT4 discontinuation in patients who were three years old or whose treatment was discontinued before, and patients with a TSH value >10 mIU/mL after being off-treatment were evaluated as permanent hypothyroidism and LT4 therapy was reinitiated in these patients. Patients whose fT4 and TSH values remained within normal limits for six months after LT4 withdrawal were considered transient hypothyroidism. Free T4 and TSH were measured again at the end of the second week in cases with normal fT4 and serum TSH <20 mIU/mL in the neonatal period. Cases whose TSH value decreased below 10 mIU/mL and LT4 treatment was not started were accepted as transient neonatal hyperthyrotropinemia.

BMI was calculated using Bodyweight (kg)/Height^2^ (m^2^). The evaluation of height, weight, head circumference, and BMI SDS was made according to the data prepared by Neyzi et al. for Turkish children [12]. Obesity was defined as a BMI of more than +2 SDS for age and sex in all subjects [12]. In all cases, short stature was defined as a height of <-2 SDS according to their age and sex [12]. The fT4 (ng/dL), fT3 (pg/mL), and TSH (mIU/L) levels were measured using the radioimmunoassay method. Agenesis, ectopia or hypoplasia reported by USG was accepted as dysgenesis, and volume increase was accepted as dyshormonogenesis after evaluating together with the clinical and laboratory findings.

TSH, fT3, and fT4 levels were measured by 2-chamber 2-step enzymatic immunoassay methods using a Beckman Coulter DxI 800 device (Beckman Coulter Inc., CA, USA). Our laboratory uses the reference values provided in the Beckmann Coulter TSH and fT4 kits, which are TSH lower and upper limits 0.35–5.6 mIU/mL, and fT4 lower and upper limits were 0.61–1.32 ng/dL. The fT3 normal range was 1.9–4.2 pg/mL.

### 2.1. Statistical methods

Statistical evaluation was done using SPSS (Statistical Package for Social Science), version 17 (IBM Inc., Armonk, NY, USA). Whether the quantitative variables were normally distributed or not was tested with the one-sample Kolmogorov Smirnov test. The Mann-Whitney U test was used to compare data that were skewed, and the chi-square test was used to compare categorical data between groups. Descriptive statistics for the data are given as median (minimum-maximum) for skewed parameters and mean±SDS for normally-distributed parameters. A receiver operator characteristics (ROC) analysis was applied to determine the cut-off of the LT4 dose at the time of treatment discontinuation as a predictive criterion for distinguishing permanent and transient CH, and the sensitivity and specificity values were calculated for this threshold value. A p-value of <0.05 was considered significant.

## 3. Results

**Figure 1:**
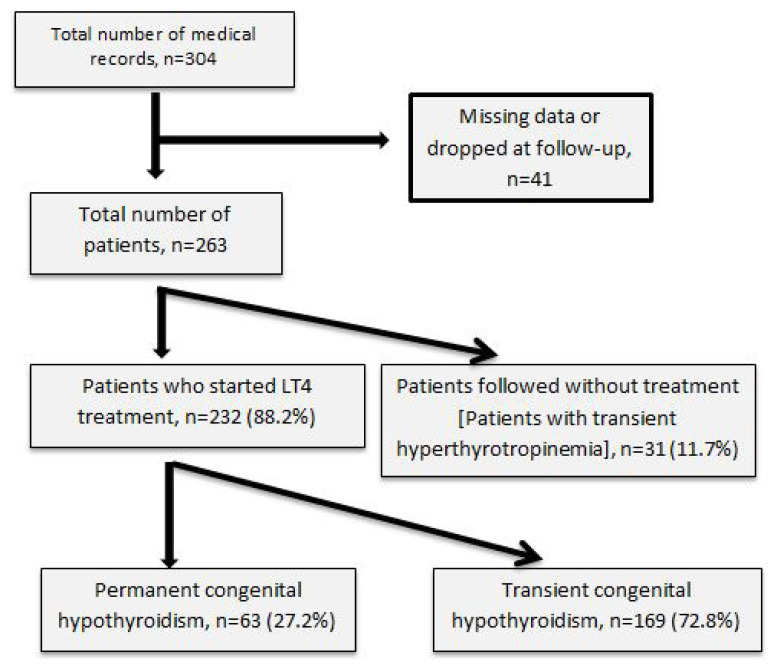
Flow diagram of the follow-up study.

A total of 304 patients’ medical records were scanned. However, 41 patients were excluded due to insufficient data, leaving 263. Thirty-one of these followed-up patients were excluded due to transient hyperthyrotropinemia. The remaining 232 patients started on L-thyroxine (LT4) were analyzed. Flow diagram of the follow-up study is shown in Figure 1.

Of the cases, 108 (46.6%) were female, and 124 were male. The mean fT3 level at the time of diagnosis was 3.3 ± 1.5 ng/dL, the fT4 level was 0.78 ± 0.4 ng/dL, and the mean serum TSH level was 54.6 ± 34.6 mIU/mL. There was no difference between the groups with permanent and transient CH in terms of sex, gestational week, birth weight, age at diagnosis (days), and age at onset of treatment (days). However, significant serum fT3, fT4, and TSH differences were found in patients with permanent and transient CH. Main characteristics of babies with permanent and transient CH are shown in Table 1. In the whole cohort the mean age at diagnosis was median 14 days with a range of 4–90 years, while the mean age at diagnosis was 17.3 ± 0.9 days in term babies (37 completed gestational weeks or more), and this rate was 21.4 ± 4.3 days in preterms. While thyroid USG was normal in all patients in the transient elevated neonatal TSH group, agenesis in 18 patients (28.6%), ectopic thyroid in four patients (6.3%), hypoplasia in three patients (4.8%), and a hemiagenesis in one patient (1.6%) were found in the permanent hypothyroidism group (Table 1). Dyshormonogenesis was the most common etiology in both transient and permanent CH. Distribution of patients with permanent and transient congenital hypothyroidism according to their etiology are shown in Table 2 and Table 3.

Mean LT4 doses at baseline, 6^th^, 12^th^, 18^th^, 24^th^, 30^th^, and 36^th^ months were lower in patients with transient CH than in permanent CH. Follow-up and treatment data of permanent and transient congenital hypothyroidism cases are shown in Table 4. When the use of iodized solution in umbilical care of the baby in the neonatal period was examined, the use of iodinated solution was found in nine cases. Transient CH was found in six of these cases and transient hyperthyrotropinemia of the newborn was found in the remaining three. Treatment was discontinued at six months in seven (4.1%), at 12 months in 29 cases (17.2%), at 18 months in 15 cases (8.9%), at 24 months in 51 cases (30.2%), at 30 months in six cases (3.6%), and at 36 months in 61 cases (36.1%). LT4 treatment was discontinued before 36 months in 108 (63.9%) of the cases with transient CH.

At the time of diagnosis, mean height SDS was significantly greater in patients with permanent CH than in transient CH. However, there was no significant difference at the end of three years. At the time of diagnosis, the head circumference SDS was higher in patients with permanent CH, but this difference disappeared at the end of one year. Height SDS, weight SDS, BMI SDS, and head circumference SDS in cases referred because of suspected congenital hypothyroidism are shown in Table 5. Only one patient had short stature at the time of diagnosis, and this patient with transient CH had normal height at the age of three years. At the end of 3 years of age, short stature was detected in three cases. Of these cases, two had a history of small for gestational age (SGA) and were diagnosed with permanent CH, and one was diagnosed with transient CH and idiopathic short stature.

**Figure 2:**
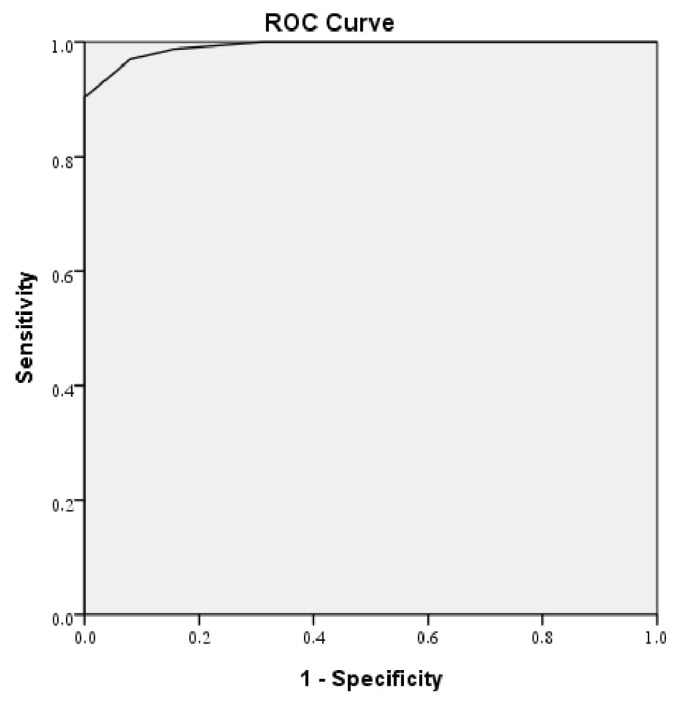
The best cut-off point was determined using the Youden index. The best cut-off point for predicting permanent status was an LT4 cut-off dose of ≥1.45 mcg/kg/day at the time of beginning a trial off replacement LT4. ROC: receiver operating characteristics

As the best criterion for distinguishing between permanent and transient hypothyroidism, the threshold of the LT4 dose at the time of discontinuation was ≥1.45 mcg/kg/day by ROC analysis, the area under the curve (AUC) was 0.992 (95% confidence interval (CI) = 0.985–1.000), which was statistically significant (p < 0.001). The diagnostic sensitivity was 100% for this cut-off value, and the specificity was 90.4% as shown in Figure 2.

## 4. Discussion

Treatment of CH is easy, inexpensive, and highly effective [13]. Thyroid hormone is a crucial hormone for brain development and functions in the fetal and postnatal periods [14]. Therefore, diagnosis and initiation of treatment in the first two weeks after birth are critical for preventing irreversible mental retardation and motor dysfunction [14,15]. Distinguishing permanent and transient conditions in patients with suspected CH will prevent unnecessary treatment in transient patients and prevent inadequate treatment in permanent patients.

As a result of the screening program conducted in Turkey between 2008–2010, the frequency of CH was 1/650, and the female/male ratio was similar [9]. In our study, no difference was found between males and females. However, consistent with the literature, males were more common in the group diagnosed with transient CH [10,16,17]. With the introduction of neonatal screening programs, the age at diagnosis has decreased significantly. In studies, the mean age at diagnosis was reported as 11–12 days, and in Turkey, it was 15.7 days in 2010 [9,18,19]. The main reason why the age at diagnosis was approximately 3 days later than Turkey’s national data might be related to the delayed TSH elevation of the premature babies included in the study [11]. In our study, the mean age at diagnosis was 17.3 days in term babies, while this rate was 21.4 days in preterms. In addition, the socio-economic status of the people living in the region, the large population from rural areas, poor transportation, the distance from the health service, and the problem in accessing the screening results affect this delay.

In our clinic, 72.8% of the cases were diagnosed with transient CH and some cases taking LT4 improved before the age of three years. Notably, approximately 65% of transient CH cases discontinued LT4 replacement therapy before the age of three years at a median duration of 24 months. Kara et al. reported that in 70% of transient CH cases, the treatment was discontinued before the age of three and with a median age of 19 months [10]. Although permanent CH was previously reported more frequently in the etiology of CH, an increase in the frequency of transient CH has attracted attention with the widespread use of screening programs in recent years. In various studies, transient CH has been reported to make up between 36.5% and 79.4% of referrals [10,17,20,21]. In the study of Park et al., the frequency of transient CH was 65% [22]. In various studies conducted in Turkey, the frequency of transient CH has been reported between 30%–75% [10,17,23–25]. In our study, the frequency of transient CH was 72.8%, which is at the high end of the reported ranges.

Among the reasons why the frequency of transient CH differed between studies, factors such as differences in inclusion criteria (term/preterm), use of different TSH threshold values to define transient hypothyroidism, maternal iodine deficiency, iodine overload in the mother/baby (often iatrogenic), or the transmission of TSH-blocking antibodies to the fetus due to autoimmune thyroiditis in the mother may play important roles [2,11,15,26,27]. In a study conducted across Turkey and investigating iodine status, it was shown that iodine deficiency is still evident in the Eastern Anatolia region of Turkey, where the study center is located [26]. Therefore, we believe that iodine deficiency may have an important role in the etiology of transient hypothyroidism in our cases, and could well be associated with the enlarged thyroid glands found in nearly three quarters of the transient cases. In our study, the frequency of maternal autoimmune thyroiditis was unknown and the frequency of transient hypothyroidism due to maternal TSH receptor-blocking antibody was not assessed as these antibodies were not measured in the mother and baby. Our study observed that hypothyroidism was transient in six of nine cases that were probably due to use of an iodinated solution in umbilical care. However, in our study, iodine overload could not be proven because the urine iodine level was not checked in infants at the time of diagnosis.

The most common (61%–85%) cause of permanent CH is accepted to be thyroid dysgenesis [10,27], while the second most common is dyshormonogenesis (10%–39%) [16,28]. Since enzyme deficiency in dyshormonogenesis is inherited in an autosomal recessive fashion, consanguineous marriage between mother and father is important in the medical history of these patients, as well as family history of CH, especially in older siblings [28]. In our study, the frequency of permanent CH was 27.2%, and in terms of etiology, thyroid dysgenesis was found in 40.0% of the cases, dyshormonogenesis in 49.2%, prematurity in 6.3%, and central CH in 4.8%. In our study, the history of consanguineous marriage in 20% of CH cases diagnosed with dyshormonogenesis suggests that Mendelian inheritance should be investigated in the etiology.

It is very important to distinguish permanency and transiency in CH cases, both to prevent unnecessary treatment for transient cases and to ensure that permanent hypothyroidism cases receive appropriate treatment. Various criteria have been investigated to predict transient and permanent hypothyroidism in cases where scintigraphy could be performed for etiological evaluation at the time of diagnosis or in cases with the ectopic thyroid gland in scintigraphy. Among these criteria, the most investigated are serum fT4, TSH levels, and LT4 dose at diagnosis and follow-up. Reports vary on this subject. Although it has been reported that fT4 at the time of diagnosis may be effective in determination in some studies and TSH in others, there are also studies stating that both are not determinative [8,17,24,25,29,30]. In their study, Tamam et al. reported that TSH levels were significantly higher and free T4 levels were significantly lower in the permanent hypothyroidism group at the time of diagnosis compared to the transient hypothyroidism group [30]. In our study, fT4 and fT3 levels were significantly lower in permanent CH, while TSH was higher at diagnosis. These results suggested that, contrary to most studies, fT4, fT3, and TSH levels at the time of diagnosis can be a criterion for distinguishing permanent and transient hypothyroidism.

Most studies reported that the LT4 dose of the permanent group was higher than the transient group at LT4 withdrawal [10,17,20,24]. In our study, the dose of LT4 at the time of discontinuation of treatment (which was determined as a predictive criterion for the differential diagnosis of permanent and transient hypothyroidism) was evaluated. The best cut-off point for predicting permanent status was determined as an LT4 cut-off dose of ≥1.45 mcg/kg/day. When this threshold value was taken, the probability of predicting the permanent state was 81% with a specificity of 90%.

If CH is not treated, it causes irreversible mental retardation and reversible short stature [4]. Neonatal screening programs for CH have successfully improved both cognitive and growth outcomes in recent years [4,11]. In our study, while the mean height SDS was significantly higher in patients with permanent CH at the time of diagnosis than in transient CH, no difference was found between them at the end of three years. While the head circumference SDS at the time of diagnosis was higher in patients with permanent CH, this difference disappeared at the end of one year. At the end of three years, short stature was detected in only three patients (1.2%). While two of these patients were SGA, one was diagnosed with idiopathic short stature. This situation shows once again how important the treatment for physical and mental development in patients with CH is.

The most important limitation of our study is that it was designed retrospectively. In addition, maternal and baby urinary iodine levels were not checked, which will probably have affected thyroid functions, especially given that our region is a known area of iodine deficiency. Furthermore, transplacental transmission of maternal thyroid autoantibodies and maternal drugs potentially affecting thyroid functions were not evaluated. Another limitation is the inclusion of patients diagnosed in other centers, although thyroid function tests are performed with a similar method and reference intervals are similar.

In conclusion, the frequency of transient CH was 72.8%. Furthermore, LT4 dose at the time of discontinuation of treatment was shown to be predictive of the permanence of CH versus transient thyroid dysfunction while fT4 and TSH levels at the time of diagnosis were also significant among the predictive criteria in the differential diagnosis of transient and permanent CH.

## Figures and Tables

**Table 1:** Main characteristics of babies with permanent and transient CH.

Characteristics	Transient CH (n: 169)	Permanent CH (n: 63)	p
Sex ratio (Female/male)	1:1	0.8:1	
Birth weight, g	3.099 ± 600	3.165 ± 468	0.811
Mode of delivery (normal birth: C/S ratio)	0.45:1	0.62:1	0.170
Consanguineous marriage, n (%)	13 (22.4)	19 (12.7)	0.065
CH history, n (%)	13 (22.0)	11 (7.6)	0.050
Birth weight <2500 g, n (%)	7 (11.5)	12 (7.5)	0.245
Gestational age <37 weeks, n (%)	6 (9.8)	15 (9.4)	0.548
Admission age, days	18.0 ± 14.1	18.3 ± 15.5	0.928
Treatment initiation age, days	18.5 ± 14.3	18.4 ± 15.5	0.901
TSH (μU/mL)	74.9 ± 36.7	47.3 ± 30.8	<0.001
fT4 (ng/dL)	0.53 ± 0.39	0.88 ± 0.33	<0.001
fT3 (ng/dL)	2.0 ± 1.6	3.9 ± 1.1	<0.001
Thyroid imaging (USG), n	63	169	
Normal	37 (58.7)	162 (95.8%)	<0.001
Abnormal	26 (41.3%)	7 (4.2%)	
Agenesis	18 (28.6%)	-	
Ectopic gland	4 (6.3%)	-	
Hypoplastic gland	3 (4.8%)	7 (4.2%)	
Hemiagenesis	1 (1.6%)		

CH: Congenital hypothyroidism, TSH: Thyroid-stimulating hormone, fT4: Free thyroxine, fT3: Free triiodothyronine, C/S: Cesarean section, USG: Ultrasonography.

**Table 2:** Distribution of patients with permanent congenital hypothyroidism according to their etiology.

	Male (n = 31)	Female (n = 32)	Total (n = 63)
Thyroid agenesis	6 (19.4%)	12 (37.5%)	18 (28.6%)
Ectopic thyroid	2 (6.5%)	2 (6.3%)	4 (6.3%)
Thyroid hypoplasia	2 (6.5%)	1 (3.1%)	3 (4.8%)
Central hypothyroidism	2 (6.5%)	1 (3.1%)	3 (4.8%)
Prematurity	3 (9.7%)	1 (3.1%)	4 (6.3%)
Dyshormonogenesis	16 (51.6%)	15 (46.9%)	31 (49.2%)
Total	31 (100%)	32 (100%)	63 (100%)

**Table 3:** Distribution of patients with transient congenital hypothyroidism according to their etiology.

	Male (n = 93)	Female (n = 76)	Total (n = 169)
Iodine exposure	4 (4.3%)	2 (2.6%)	6 (3.6%)
Antithyroid drug use in the mother	2 (2.2%)	1 (1.3%)	3 (1.8%)
Prematurity	10 (10.8%)	6 (7.9%)	16 (9.5%)
Dyshormonogenesis	77 (82.8%)	67 (88.2%)	144 (85.2%)
Total	93 (100%)	76 (100%)	169 (%)

**Table 4:** Follow-up and treatment data of permanent and transient congenital hypothyroidism cases.

Parameter	Permanent CH		Transient CH		p
Follow-up time, months	≥36		≥36		
Median age of treatment discontinuation, months	-		24 (6–36)		
L-thyroxine dose (μg/kg/day), n	n = 63		n = 169		
Starting dose	63	10.0 ± 3.4	169	7.3 ± 2.8	<0.001
Dose at 6 months	51	3.9 ± 1.2	148	2.1 ± 0.8	<0.001
Dose at 12 months	52	3.0 ± 0.8	142	1.5 ± 0.5	<0.001
Dose at 18 months	52	2.8 ± 0.7	113	1.3 ± 0.4	<0.001
Dose at 24 months	53	2.6 ± 0.7	97	1.2 ± 0.4	<0.001
Dose at 30 months	50	2.5 ± 0.6	56	1.1 ± 0.3	<0.001
Dose at 36 months	51	2.4 ± 0.5	52	0.9 ± 0.3	<0.001
At withdrawal time of L-thyroxine	31	1.4 ± 0.5	169	1.1 ± 0.3	<0.001

Minimum and maximum (min-max) values are given in parentheses.

**Table 5:** Height SDS, weight SDS, BMI SDS, and head circumference SDS in cases referred because of suspected congenital hypothyroidism.

	Total (n = 232) (min-max)	Permanent CH (n = 63)	Transient CH (n = 169 )	p
Sex				0.260
Female, n(%)	108 (46.8)	32 (50.8)	76 (45.0)	
Male, n(%)	123 (53.2)	31 (49.2)	93 (55.0)	
At diagnosis				
Height SDS	0.03 (−2.02/2.2)	0.33 ± 0.75	0.01 ± 0.68	0.026
Weight SDS	0.26 (−1.8/2.85)	0.42 ± 0.82	0.21 ± 0.84	0.222
BMI SDS	0.33 (−2.3/3.0)	0.26 ± 1.11	0.30 ± 0.92	0.840
HC SDS	−1 (−1.93/1.98)	0.07 ± 0.68	−0.25 ± 0.74	0.029
1 year old				
Height SDS	−0.30 (−2.1/3.2)	−0.31 ± 0.88	−0.20 ± 0.86	0.498
Weight SDS	0.12 (−2.0/2.5)	0.23 ± 0.81	0.02 ± 0.87	0.209
BMI SDS	0.39 (−3.0/2.8)	0.64 ± 0.76	0.23 ± 0.94	0.024
HC SDS	0.76 (−1.8/2.06)	0.20 ± 0.73	0.09 ± 0.77	0.448
2 years old				
Height SDS	−0.23 (−2.5/2.7)	−0.08 ± 1.23	−0.19 ± 0.87	0.617
Weight SDS	0.09 (−2.15/2.8)	0.06 ± 1.19	0.15 ± 0.90	0.694
BMI SDS	0.2 (−2.6/3.2)	0.17 ± 1.11	0.27 ± 1.01	0.645
3 years old				
Height SDS	−0.11(−2.8/3.02)	−0.13 ± 1.3	−0.17 ± 0.73	0.866
Weight SDS	0.09 (−3.1/3.30)	0.14 ± 1.4	0.05 ± 0.91	0.742
BMI SDS	0.20 (−2.61/3.2)	0.28 ± 1.10	0.20 ± 0.96	0.747

SDS: Standard deviation score, BMI: Body mass index, HC: Head circumference. Data are given as means. Minimum and maximum (min/max) values are given in parentheses.
